# An Unusual Cause of Coronary Occlusion During an Abdominal Aortic Aneurysm Repair: Reperfusion With Diagnostic Angiography Only

**DOI:** 10.7759/cureus.39610

**Published:** 2023-05-28

**Authors:** Omkar Betageri, Hassan Ashraf, Adam Hafeez, Richard Kerensky, Thomas S Huber, Michael Massoomi

**Affiliations:** 1 Cardiology, Maine Medical Center - Tufts University, Portland, USA; 2 Cardiology, University of Texas at Houston, Houston, USA; 3 Cardiology, University of Florida College of Medicine, Gainesville, USA; 4 Vascular Surgery, University of Florida College of Medicine, Gainesville, USA

**Keywords:** abdominal aortic aneurysm, acute coronary syndrome, coronary angiography, peri-operative, myocardial infarction

## Abstract

We present a unique case of a type I peri-operative myocardial infarction during an extensive abdominal aortic aneurysm repair occurring due to the occlusion of a severe stable ostial plaque stenosis by a small overlying thrombus. During coronary angiography, the thrombus was dislodged by the diagnostic catheter which restored normal flow without stent placement. We demonstrate a care approach that was carefully arrived upon through multidisciplinary management with vascular surgery and anesthesiology colleagues.

## Introduction

Myocardial infarction is defined as myocardial necrosis evidenced by cardiac-specific biomarkers in a clinical setting consistent with myocardial ischemia. Peri-operative myocardial infarctions (PMI) in non-cardiac surgeries have an incidence of 16%, and greater than 50% are silent [1-2]. ECG changes may be subtle and transient in these patients and more commonly present as non-Q wave infarction [2]. PMI can occur via the type 1 and type 2 mechanisms. Type I PMI is hallmarked by increased coronary shear stress leading to plaque rupture and the effects of post-operative hypercoagulability. Conversely, type II PMI is driven by a supply and demand mismatch and clinically manifested as prolonged ST depression on ECG.

We present a unique case of a type I PMI during an extensive abdominal aortic aneurysm (AAA) repair not due to a rupture of an unstable coronary plaque with subsequent thrombotic occlusion but an occlusion of severe stable plaque stenosis by a small overlying thrombus. The diagnosis was made intraoperatively with an ECG and transesophageal echocardiogram. During coronary angiography, the thrombus was dislodged by the diagnostic catheter which restored normal flow.

## Case presentation

A 71-year-old male with a significant medical history of peripheral vascular disease, hypertension, and ongoing tobacco use was referred to our institution for the repair of an incidentally discovered 7 cm juxtarenal AAA as well as bilateral iliac artery occlusion. Three months prior, he underwent pre-operative cardiac testing with a nuclear myocardial perfusion scan which was abnormal and a subsequent coronary angiogram which was reported as non-obstructive coronary artery disease. He was started on clopidogrel for medical management.

**Figure 1:**
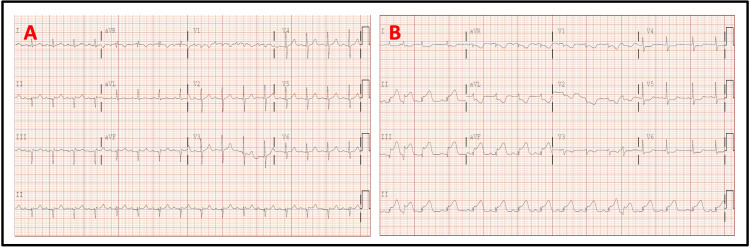
ECG tracings Tracing A: Baseline ECG (one-week pre-surgery) Tracing B: Post-surgical ECG

He underwent surgery under general endotracheal anesthesia and was heparinized with 100 U/kg prior to vascular dissection. A 20 x 10 Hemashield™ graft was successfully used for the repair of a 7 cm aortic aneurysm. He was found to have profound iliac disease for which he had successful endarterectomy on the right iliac artery. Due to the profound distal disease on the left side, he had an 8 mm Hemashield™ graft sown to the left limb of his aortic graft and tunneled through the groin to the deep femoral artery. The superficial femoral artery was anastomosed to the hood of the iliofemoral limb, and flow was restored to both distal lower extremities. The case was complicated by 4.2 L of blood loss, and the patient received 8L crystalloid, 750 cc 5% albumin, 5 U fresh frozen plasma, 2 U packed red blood cells, and 1 unit of platelets. The heparin was reversed with protamine prior to surgical wound closure. While the abdominal wound was being closed, the patient became hypotensive, and telemetry showed ST elevations. An ECG subsequently obtained confirmed ST elevations in the inferior leads (Figure 1).

Intraoperative transesophageal echocardiography (TEE) showed preserved biventricular function with subtle inferior hypokinesis on the transgastric short axis view, without pericardial effusion or significant valvular pathology. After discussion with anesthesia and surgery, a decision was made to emergently transfer the patient to the cardiac catheterization lab due to high suspicion of type I PMI. Prior to transfer, he developed ventricular fibrillation requiring cardiopulmonary resuscitation with defibrillation twice prior to the return of spontaneous circulation.

In the catheterization lab, right radial access was obtained, and a Prelude IDEAL™ 5/6Fr 11cm sheath was placed. Through this, a Terumo Medical Ikari left 3.75 5Fr catheter was inserted over a Terumo Medical Runthrough® 0.014” x 180 cm wire. Engagement of the catheter with the left coronary artery revealed mild non-obstructive coronary artery disease of the left anterior descending (LAD) artery (Videos 1-2).

**Video 1:** An Ikari 5Fr guiding catheter is shown engaging the left coronary artery in the right anterior oblique (RAO) caudal projection. The patient has mild non-obstructive coronary artery disease

**Video 2:** An Ikari 5Fr guiding catheter is shown engaging the left coronary artery in the RAO cranial projection. The patient has mild non-obstructive coronary artery disease in the LAD artery

The right coronary artery (RCA) was subsequently engaged. This was made challenging by notable ostial RCA calcification and stenosis (Video 3).

**Video 3:** An Ikari 5Fr guiding catheter is shown engaging the RCA in the left anterior oblique (LAO) cranial projection. Initially, there was difficulty cannulating the right coronary ostium, requiring brief contrast administration to help visualize the RCA ostium. Suddenly, the RCA appeared and there appeared to be a severe calcified ostial lesion along with a diffuse disease RCA with thrombolysis in myocardial infarction (TIMI) II flow

The initial angiogram after engagement showed TIMI II flow (Video 4), and the subsequent angiogram showed improvement to nearly TIMI III (Video 5).

**Video 4:** An Ikari 5Fr guiding catheter is shown engaging the RCA in the RAO projection with TIMI III flow through the RCA

**Video 5:** An Ikari 5Fr guiding catheter is shown engaging the RCA in the LAO projection with TIMI III flow through the RCA

**Figure 2:**
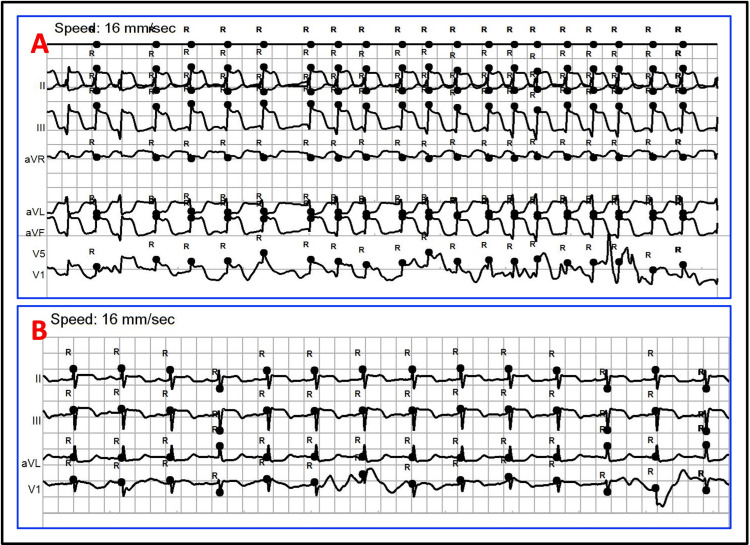
Intra-catheterization ECG tracings Tracing A: Prior to RCA engagement Tracing B: After RCA engagement

During this time, ST elevations began to subside and revert to a near baseline isoelectric state (Figure 2). After discussion with vascular surgery, conservative treatment without stent placement was decided upon due to bleeding risk. He was medically treated with heparin and clopidogrel, remained stable in the cardiac care unit without recurrence of ST segment changes, and ultimately was discharged in stable condition. His transthoracic echocardiography obtained three days after surgery showed preserved biventricular function without wall motion abnormalities.

## Discussion

We present a unique case of a peri-operative ST-elevation myocardial infarction caused by an occlusion of severe stable plaque stenosis with an overlying thrombus which was successfully dislodged unPerioperativeintentionally with catheter manipulation during an angiogram. A review of a pre-operative coronary angiogram performed three months before surgery confirmed the presence of severe calcified stenosis that was not appreciated by the performing angiographer (Videos 6-8).

**Video 6:** Pre-operative coronary angiogram of the RCA in the LAO cranial projection showing severe calcific ostial coronary artery disease

**Video 7:** Pre-operative coronary angiogram of the RCA in the posterior-anterior cranial projection showing severe calcific ostial coronary artery disease

**Video 8:** Pre-operative coronary angiogram of the RCA in the RAO cranial projection showing severe calcific ostial coronary artery disease

As a result, no revascularization was conducted prior to the patient's elective aortic aneurysm repair. We suspect that the ostial location of the lesion may have been a primary contributing factor to the uncommon scenario of successful restoration of coronary flow during the ST-elevation myocardial infarction. Ostial lesions are typically fibrotic and stable with less relative lipid content compared to distal lesions making them less prone to rupture [3]. The difficulty in engaging the ostium of the RCA and subsequent improvement from TIMI II to TIMI III flow after engaging the artery suggests that the occlusion was due to overlaying material on top of a known high-grade lesion and not due to a plaque rupture event. Intracoronary imaging modalities, such as intravascular ultrasound (IVUS) and optical coherence tomography (OCT), were unable to be used given the acuity of the case but would be critical tools for the characterization of the coronary artery lesion (e.g., thrombus vs calcific stenosis, plaque composition).

Other pathophysiologic considerations likely to have contributed to the PMI include peri-operative hypotension, multifactorial related to hemorrhagic shock, cardiac arrest, and anesthetic-related vasoplegia. It is worth noting that the patient was on a stable dose of vasopressor support during the coronary angiography without any intraprocedural hemodynamic compromise. Furthermore, it is important to consider catheter-associated spasms of the RCA within the appearance of ostial coronary artery stenosis. Notably, the severe ostial stenosis improved with contrast administration rather than intracoronary nitroglycerin, which was not used due to ongoing shock.

PMI pathophysiology

Our case describes a type 1 myocardial infarction not due to rupture of an unstable coronary plaque with subsequent thrombotic occlusion but possibly due to occlusion of severe stable plaque stenosis by a small overlying thrombus that was dislodged by the diagnostic catheter. The ostial location of the lesion led to this uncommon scenario for successful restoration of coronary flow during ST-elevation myocardial infarction. Furthermore, unstable atherosclerotic plaques are often minimally diseased prior to occlusion, often with <50% stenosis [4]. Additionally, stable severe stenoses are often not the site of thrombotic occlusion resulting in acute myocardial infarction [5].

Patients with underlying coronary artery disease are at risk of peri-operative plaque rupture due to increased coronary shear stress with tachycardia and hypertension. Additionally, PMI often occurs at or near the end of the surgery, driven by a rise in catecholamines during skin closure and sympathetic discharge after emergence from anesthesia [6]. Post-operative patients are also in a hypercoagulable state due to increased platelet activation and decreased fibrinolytic activity [7].

PMI risk reduction

Prophylactic medical therapy remains an option for PMI, yet with overall equivocal results. The peri-operative ischemic evaluation trial showed a 27% lower rate of myocardial infarction in patients receiving metoprolol succinate 30 days prior to surgery vs placebo but a statistically higher rate of death (31%) and stroke (100%) [8]. Beta-blockade can worsen hypotension and counteract the physiological response to increased cardiac output in the setting of active bleeding, anemia, or infection. As such, the use of beta-blockers as prophylactic therapy remains an area of contention. However, beta-blockers should be continued in patients who are receiving them as a treatment for angina, hypertension, arrhythmias, or other ACC/AHA Class I guideline indications. Statin therapy has been associated with reduced peri-operative and long-term cardiac complications and is advised to be continued peri-operatively [3]. Other therapies such as calcium channel blockers and alpha-2 agonists have not been shown to be associated with a substantive reduction in PMI incidence or mortality [9].

Prophylactic preoperative coronary revascularization, primarily by coronary artery bypass grafting, has been studied and has been associated with mostly negative results. While one meta-analysis has shown improved outcomes in patients undergoing prophylactic coronary revascularization, several prospective randomized control trials did not show benefit [10].

PMI management and case analysis

PMI without ST changes or Q waves can be difficult to diagnose and may be detected by the presence of hemodynamic instability, tachycardia, and pulmonary congestion. In our case, the patient had ST-segment elevations on telemetry confirmed with a 12-lead ECG. Additional tests which can be performed for the suspicion of PMI include troponins, complete blood count, and arterial blood gas.

In patients who have definitive evidence of ST-segment elevation, as in our case, expeditious cardiology consultation for further evaluation is necessary. While additional analysis with TTE/TEE may take place, this must occur concurrently with a thoughtful discussion regarding coronary angiography and reperfusion. Interestingly, no guidelines currently exist regarding anticoagulation in post-operative ST-elevation myocardial infarction without a stenting setting, and this remains an area for further investigation.

## Conclusions

We share a unique case of a type 1 PMI caused not by rupture of an unstable coronary plaque with subsequent thrombotic occlusion but possibly by the presence of stable ostial calcified stenosis on which an overlying thrombus caused acute occlusion. This occlusion may subsequently have been dislodged by a standard guiding catheter, and flow was restored without further coronary intervention. Important pathophysiologic considerations in the context of PMI include perioperative hypotension and catheter-induced spasm. Intravascular imaging modalities such as IVUS or OCT should be used whenever feasible to clarify pathophysiologies such as thrombus or calcified plaque.
